# From incivility to outcomes: tracing the effects of nursing incivility on nurse well-being, patient engagement, and health outcomes

**DOI:** 10.1186/s12912-024-01996-9

**Published:** 2024-05-13

**Authors:** Nourah Alsadaan, Osama Mohamed Elsayed Ramadan, Mohammed Alqahtani

**Affiliations:** 1 College of Nursing, Jouf University, Sakaka, 72388 Saudi Arabia; 2 Department of Nursing, College of Applied Medical Sciences, King Faisal University, Alahsa, Saudi Arabia

**Keywords:** Nursing incivility, Workplace mistreatment, Nurse stress, Patient engagement, Health outcomes, Workplace intervention and healthcare quality

## Abstract

Nursing incivility, defined as disrespectful behaviour toward nurses, is increasingly recognized as a pressing issue that affects nurses’ well-being and quality of care. However, research on the pathways linking incivility to outcomes is limited, especially in Saudi hospitals. Methods: This cross-sectional study examined relationships between perceived nursing incivility, nurse stress, patient engagement, and health outcomes in four Saudi hospitals. Using validated scales, 289 nurses and 512 patients completed surveys on exposure to incivility, stress levels, activation, and medication adherence. The outcomes included readmissions at 30 days and satisfaction. Results: More than two-thirds of nurses reported experiencing moderate to severe workplace incivility. Correlation and regression analyzes revealed that nursing incivility was positively associated with nursing stress. An inverse relationship was found between stress and patient participation. Serial mediation analysis illuminated a detrimental cascade, incivility contributing to increased nurse stress, subsequently diminishing patient engagement, ultimately worsening care quality. Conclusions The findings present robust evidence that nursing incivility has adverse ripple effects, directly impacting nurse well-being while indirectly affecting patient outcomes through reduced care involvement. Practical implications advocate for systemic interventions focused on constructive nursing cultures and patient empowerment to improve both healthcare provider conditions and quality of care. This study provides compelling information to inform policies and strategies to mitigate workplace mistreatment and encourage participation among nurses and patients to improve health outcomes.

## Introduction

Nursing, a cornerstone of the healthcare system, plays an indispensable role in patient care and the broader health landscape [1, 2]. This noble profession encompasses not only the administration of treatments and medications but also the provision of emotional support and education to patients and their families [3, 4]. Nurses are often the primary point of contact for patients, which makes their role crucial in shaping patient experiences and outcomes [5]. The diverse responsibilities, from bedside care to patient advocacy, emphasize the multifaceted nature of nursing and its critical impact on the delivery of healthcare [6, 7]. The work environment in which nurses work is crucial for both their well-being and their ability to provide quality care [6, 8]. A positive and supportive environment not only improves job satisfaction and retention among nurses but also directly influences patient safety and quality of care [9, 10]. Factors such as teamwork, communication, and organizational culture play an important role in shaping this environment [11]. In contrast, negative elements within the workplace can lead to burnout, decreased job satisfaction, and potentially compromise patient care [12].

Nursing incivility, an increasingly distressing concern, encompasses disrespectful behaviours [13], that violate workplace dignity norms ranging from subtle belittling to overt hostility [2, 8]. This widespread phenomenon permeates most healthcare settings [14, 15], with up to 85% of nurses encountering this mistreatment from various sources [16], resulting in a significantly disruptive organizational climate [17]. Beyond affecting nurse well-being through adverse psychological impacts, incivility breeds poor morale, compromised performance, increased attrition, and, critically, reduced quality of patient care [18–20]. Prioritizing healthy collegial environments remains crucial for upholding both nurse wellness and optimal patient outcomes [21–23]. Furthermore, organizational factors, such as leadership, communication, and workplace culture, may play a significant role in shaping the dynamics of nursing incivility, stress, and patient outcomes [24, 25]. Investigating these factors could provide a more comprehensive understanding of the complex interplay between individual and systemic elements in the healthcare setting [26, 27]. Nursing incivility can manifest itself in various forms, including, but not limited to, belittling comments, bullying, gossip, and exclusionary tactics [11, 28]. These behaviours can originate from colleagues, superiors, patients, and their families [16, 29]. Such conduct not only undermines professional relationships [30] but also can cause psychological distress for victims, preventing their ability to perform effectively [31, 32].

Although the prevalence and nature of incivility in nursing have been well documented, there remains a significant gap in understanding its full impact [33, 34]. The impact of nursing incivility extends beyond the immediate targets, affecting multiple aspects of healthcare delivery [35, 36]. Incivility can have profound emotional consequences for nurses, leading to increased stress, burnout, and job dissatisfaction, which can compromise their ability to provide high-quality patient care [8, 19, 29]. Moreover, uncivil behaviors can strain nurse-patient interactions, potentially diminishing the quality of care and patient satisfaction [18, 37]. At an organizational level, incivility can disrupt team dynamics, contribute to higher staff turnover rates, and negatively influence the overall culture within healthcare institutions [18, 38, 39]. Furthermore, the economic implications of nursing incivility, such as costs associated with staff replacement and lost productivity due to absenteeism and presenteeism, warrant further investigation [40–42]. Examining these multifaceted impacts is crucial for developing targeted interventions and policies to mitigate the detrimental effects of incivility on nurses, patients, and healthcare organizations [43, 44].

Current literature has primarily focused on identifying forms and instances of uncivil behavior, often overlooking their deeper implications for nurses, patients, and healthcare systems. An underexplored area is the direct effect of incivility on nurses’ well-being [8]. This includes quantifying the emotional and professional toll, such as stress, burnout, and job dissatisfaction [31], which are crucial factors influencing nurse retention and mental health [45, 46].

In summary, filling these gaps through robust empirical research is crucial. Such research is essential not only to transform current anecdotal and observational understandings into data-driven insights but also to develop effective strategies to mitigate the negative impacts of incivility [47, 48]. These insights are vital to promoting a healthier, more respectful, and efficient healthcare environment, ultimately enhancing nurses’ well-being and patient care quality [3, 5]. The primary objective of this study was to investigate the impact of nursing incivility on critical aspects of healthcare care delivery. By focusing on nurse stress, patient engagement, and health outcomes (defined as 30-day readmission rates and patient satisfaction scores), the study aimed to understand how incivility in the nursing environment affects both healthcare providers and recipients.

The study was conducted within the context of the Saudi healthcare system, which has undergone significant reforms in recent years [49, 50]. The system is primarily government-funded, with a growing private-sector presence [51]. It aims to provide universal access to healthcare services for all citizens and residents, with a focus on improving quality and efficiency [52]. However, like many healthcare systems worldwide [53, 54], it faces challenges related to workforce development, patient satisfaction, and the management of complex health conditions [55]. Understanding the impact of nursing incivility within this context is crucial for informing strategies to enhance the well-being of healthcare providers and the quality of patient care.

This study examined nurse stress, a direct consequence of incivility, and its subsequent effects on patient care. Additionally, it explored how incivility in nursing influenced patient participation, a crucial factor in successful health outcomes. Finally, the study assessed the broader implications of these variables on overall health outcomes, providing valuable insights for healthcare policy and practice.

This study’s findings can influence nursing practice and patient care significantly. By demonstrating the tangible impacts of nursing incivility, the study can inform the development of targeted interventions and policies to create a more respectful and supportive work environment for nurses. This, in turn, can lead to improved patient care and outcomes. Highlighting the importance of a respectful and supportive nursing environment is a key outcome of this study. By underscoring the detrimental effects of incivility, the research advocates for a cultural shift in healthcare settings toward more positive and collaborative interactions. These changes are vital for nurses’ well-being, patient care quality, and healthcare organizations’ overall effectiveness.

## Materials and methods

### Research objectives & research hypothesis

1. Examine the relationships between nursing incivility, nurse stress (defined as emotional exhaustion and depersonalization), patient engagement (defined by patient activation levels and adherence to discharge protocols), and health outcomes (defined as 30-day readmission rates and patient satisfaction scores). H1a: Higher levels of nursing incivility will be positively associated with increased nurse stress. H1b: Higher levels of nurse stress will be negatively associated with patient engagement. H1c: Lower levels of patient engagement will be associated with poorer health outcomes.
2. Investigate how different perceived levels and types of nursing incivility, including overt (bullying, verbal abuse) and covert (gossip, exclusion) behaviours frequently reported by nurses, affect nurse stress and emotional exhaustion through a cross-sectional survey methodology.
  H2a: Overt forms of nursing incivility will have a stronger positive association with nurse stress compared to covert forms of incivility. Overt forms of nursing incivility refer to more explicit and direct forms of uncivil behaviour, such as verbal abuse, bullying, or intimidation. Covert forms of nursing incivility refer to more subtle and indirect forms of uncivil behaviour, such as gossip, exclusion, or undermining actions.
  H2b: A higher frequency of exposure to nursing incivility will be associated with higher levels of nurse stress and emotional exhaustion.

3. Evaluate how nursing incivility, nurse stress, and patient engagement (activation and adherence) impact patient health outcomes (30-day readmissions and satisfaction), mapping the relationships between these variables using multivariate regression techniques. H3a: Nursing incivility will have a direct negative effect on patient health outcomes. H3b: Nurse stress will mediate the relationship between nursing incivility and patient health outcomes. H3c: Patient engagement will mediate the relationship between nurse stress and patient health outcomes. H3d: The combined indirect effects of nurse stress and patient engagement will partially mediate the relationship between nursing incivility and patient health outcomes.

**Fig. 1:**
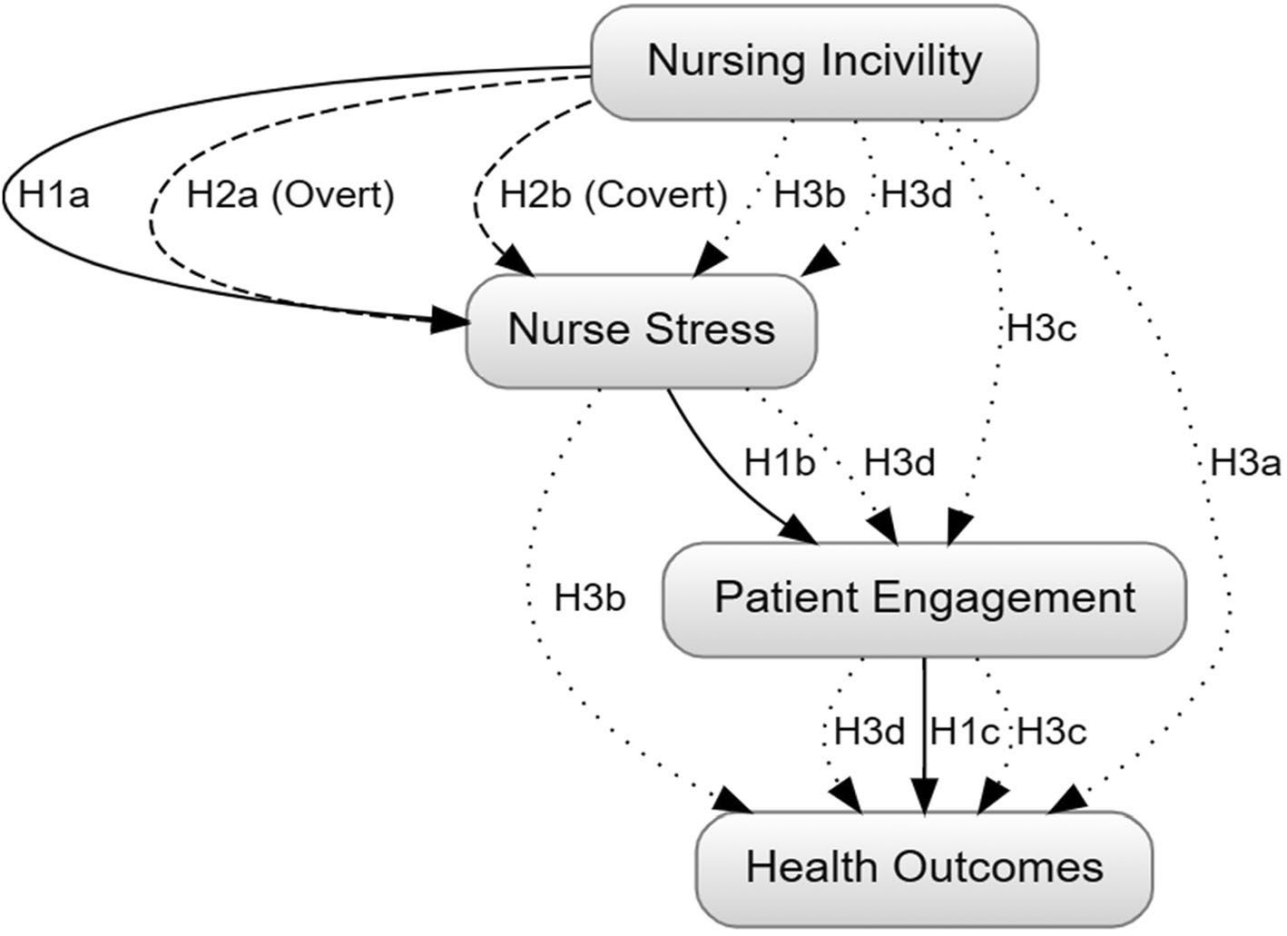
Hypothesized relationships between nursing incivility, nurse stress, patient engagement, and health outcomes

These hypothesized relationships form the conceptual foundation of our study, guiding our investigation into the complex interplay between nursing incivility, nurse well-being, patient engagement, and healthcare outcomes. By examining these relationships, we aim to provide insights into the potential cascading effects of uncivil behaviours in the nursing workplace and their ultimate impact on patient care. Figure 1 illustrates the hypothesized relationships between nursing incivility, nurse stress, patient engagement, and health outcomes. As depicted in Fig. 1, we hypothesize that nursing incivility directly influences nurse stress and patient engagement. In turn, nurse stress is expected to have an indirect effect on health outcomes, mediated by patient engagement. Additionally, we anticipate that patient engagement directly impacts health outcomes, which are operationalized as readmission rates and patient satisfaction.

The arrows in Fig. 1 are used to represent the relationships and directional hypotheses between the constructs mentioned: Nursing Incivility, Nurse Stress, Patient Engagement, and Health Outcomes. Here’s how the arrows correspond to each hypothesis:

1. *Solid Arrows indicate a direct relationship in the primary sequence of effects*:
  H1a: Nursing Incivility → Nurse Stress.
  H1b: Nurse Stress → Patient Engagement.
  H1c: Patient Engagement → Health Outcomes

2. *Dashed Arrows represent different types of incivility (overt and covert) and their effect on Nurse Stress*:
  H2a: Nursing Incivility (Overt) → Nurse Stress.
  H2b: Nursing Incivility (Covert) → Nurse Stress.

3. *Dotted Arrows show both direct and mediated paths for complex relationships*:
  H3a: Direct effect from Nursing Incivility → Health Outcomes.
  H3b: Mediated effect through Nurse Stress.
  H3c: Mediated effect through Patient Engagement.
  H3d: Combined mediation through Nurse Stress and Patient Engagement leading to Health Outcomes.

### Design

This study employed a cross-sectional correlational design to explore the relationships between nursing incivility, nurse stress, patient engagement, and health outcomes. This design involved collecting data from a defined population of nurses and patients in acute care settings simultaneously. This approach allows us to examine the associations between variables without actively manipulating any of them, providing a snapshot of the current state of these relationships.

### Settings

The study was conducted in four hospitals located in the northwest region of Saudi Arabia. The participating hospitals are large, general medical and surgical facilities, with bed capacities ranging from 200 to 500. They provide a wide range of services, including inpatient and outpatient care, emergency services, critical care units, and specialized departments such as maternity, paediatrics, and mental health treatment. The patient population served by these hospitals is diverse, encompassing individuals seeking acute care for various medical conditions as well as those managing chronic illnesses such as diabetes, cardiovascular diseases, and respiratory disorders. The hospitals cater to both urban and rural communities within the northwest region. The nursing staff in these hospitals comprises a combination of Saudi and expatriate nurses, with varying levels of experience and educational qualifications. It is important to note that the findings of this study are specifically relevant to the northwest region of Saudi Arabia and may not be generalizable to other regions or healthcare settings. The unique cultural and socioeconomic characteristics of this region should be considered when interpreting the results and their implications for nursing practice and patient care.

### Participant sample size determination

We calculated sample sizes for the nurse and patient groups to ensure statistical validity and practicality in our cross-sectional study. For the 289 nurses, we conducted a power analysis using a moderate effect size, 80% power, and a 0.05 alpha level, following the guidelines of Cohen (2013) on power analysis for behavioural sciences [56]. Although a small effect size might initially seem appropriate given the significant knowledge gap addressed by our study, the moderate effect size was chosen to maintain a balance between sensitivity and feasibility. This decision was particularly influenced by the practical challenges associated with securing a large enough sample to detect small effects within the logistical and resource constraints of our study setting. The moderate effect size was deemed most appropriate given the limited existing research on the specific relationships between nursing incivility, nurse stress, patient engagement, and health outcomes within the Saudi Arabian context, as highlighted in the introduction. Additionally, we accounted for potential variability and non-response rates for healthcare research, as suggested by Davern (2013).

The patient group required a larger sample size of 512 to accommodate greater variability and enable subgroup analyses. Patients were selected using a combination of random sampling and voluntary participation. Initially, a random sample of patients was drawn from the patient records of the participating hospitals, ensuring a representative mix of demographics, diagnoses, and hospital units. These patients were then invited to participate in the study voluntarily, which aimed to minimize selection bias while ensuring patient autonomy.

This approach also adhered to the standard power analysis methods [56] and included an upward adjustment for expected variability in patient responses, as recommended by Hulley et al. (2013) in their guidelines for clinical research [57]. Both sample sizes were further validated for feasibility within our resource constraints and specific healthcare settings, aligning with the practical considerations outlined by [58] in planning health research. In summary, the sample sizes of 289 nurses and 512 patients were determined using established statistical methods and customized to the unique aspects of our study, ensuring adequate power for reliable results. The selection process for both nurses and patients aimed to balance representativeness, statistical power, and ethical considerations, with patient selection particularly focused on combining random sampling with voluntary participation.

### Eligibility criteria

#### Inclusion criteria

Participants selected for this study were required to meet several conditions. First, they had to be registered nurses actively employed full-time, working ≥ 30 h per week, at one of the four identified healthcare hospitals. Their experience in the current institution should have spanned a minimum of six months. Furthermore, only those who could and were willing to provide informed consent were considered. Language proficiency was also crucial; Participants had to be fluent in Arabic or English to ensure they understood and completed the survey accurately. Lastly, the age bracket for eligible nurses was established between 25 and 60 years. Additionally, eligible participants must participate in direct patient care activities at least 10 h per week.

#### Exclusion criteria

Several factors led to the exclusion of potential participants from this study. Nurses who were currently not in active service, perhaps due to long-term leave or sabbatical, were not considered. We also considered the health aspect; nurses who self-declared cognitive impairments or mental health problems that could influence the accuracy of their responses were excluded. Nurses who had participated in a similar study or survey related to the topic in the last 6 months were excluded from this research. This exclusion criterion was implemented to minimize the potential influence of recent exposure to similar research questions or interventions on participants’ responses. By ensuring that a sufficient washout period had passed since any previous participation in related studies, we aimed to reduce the risk of response bias and enhance the validity of the collected data. This criterion contributes to the study’s rigour by minimizing the potential confounding effects of prior research experiences and promoting the collection of more independent and unbiased responses from participants.

### Data collection tools

In this study, we employed the following validated instruments to measure the key variables, aligning with our research objectives and hypotheses:

#### Nursing incivility scale (NIS)

The Nursing Incivility Scale (NIS) is a quantitative instrument comprising 43 items designed to measure the frequency of perceived incivility from various sources, including patients, supervisors, coworkers, and physicians, over the preceding six months [59]. The NIS includes subscales that assess various sources of incivility, such as from nurses, supervisors, physicians, and patients. The items within these subscales capture both overt and covert forms of incivility, allowing for an assessment of the frequency and severity of each type of uncivil behaviour [60].

It employs a 5-point Likert scale ranging from “Never” to “Daily” and encompasses five subscales addressing different sources of incivility: nurses, the general workplace, supervisors, physicians, and patients The Nursing Incivility Scale (NIS) doesn’t provide a direct score but rather collects data on the frequency of uncivil behaviours experienced by nurses [60, 61]. The NIS has demonstrated excellent internal reliability (Cronbach’s α > 0.90 across subscales) [56], and validity, making it well-suited for exploring the correlation between nursing incivility and nurse stress. Higher scores on the NIS subscales indicate a higher frequency of exposure to various forms of incivility from different sources.

#### Perceived stress scale (PSS)

The Perceived Stress Scale (PSS) is a 10-item self-report questionnaire that evaluates an individual’s stress appraisal over the preceding month, with a particular emphasis on predictability, control, and overload [62]. It employs a 5-point Likert scale ranging from “Never” to “Very Often.” The total PSS score typically ranges from 0 to 40 (assuming a 4-point scale), with higher scores indicating greater perceived stress and emotional exhaustion. A common interpretation guide categorizes scores as follows: 0–13 for low stress, 14–26 for moderate stress, and 27–40 for high perceived stress. The PSS has been extensively validated, exhibiting good internal reliability (Cronbach’s α = 0.78), rendering it pertinent for assessing stress levels and emotional exhaustion among nurses [63].

#### Patient activation measure (PAM)

A 13-item scale measuring patient self-efficacy in managing their health and care [64]. The Patient Activation Measure (PAM) employs a 4-point Likert scale, ranging from “Strongly Disagree” (1) to “Strongly Agree” (4), to assess the level of patient involvement in their healthcare. The raw scores from each question are summed, and this raw score is then mathematically transformed to a 0-100 scale. The final PAM score reflects the degree of a patient’s activation, with a score range of 1–46 indicating low activation, wherein patients tend to be overwhelmed and unprepared to take an active role in their health; 47–55 suggesting moderate activation, where patients are somewhat comfortable managing their health but might require assistance; 56–72 signifying high activation, with patients being comfortable in taking an active role in managing their health; and 73–100 representing very high activation, wherein patients are highly confident and skilled in managing their health [65].

#### Morisky Medication Adherence Scale (MMAS-8)

The Morisky Medication Adherence Scale (MMAS-8) is a validated 8-item self-report instrument designed to identify barriers to medication adherence [66]. It employs a binary response format (yes/no) to assess adherence issues over the past week. The MMAS-8 exhibits good internal consistency (Cronbach’s α = 0.83) and reliability, rendering it a crucial tool for evaluating patient engagement concerning medication adherence. Patients are categorized into different adherence levels based on their cumulative score ranging from 0 to 8, with a score of 8 indicating high adherence (likely following medication instructions), scores of 6 or 7 suggesting medium adherence (potential for missed medications), and scores below 6 signifying low adherence (high risk of not following instructions) [67].

#### Hospital Consumer Assessment of Healthcare Providers and systems (HCAHPS)

The Hospital Consumer Assessment of Healthcare Providers and Systems (HCAHPS) is a survey instrument and data collection methodology to measure patients’ perceptions of their hospital experience [68]. The survey contains 29 questions about the recent hospital stay of patients, including communication with nurses and doctors, hospital staff responsiveness, cleanliness and quietness of the hospital environment, communication about medications, discharge information, overall hospital rating, and whether they would recommend the hospital [69]. The survey is administered to a random sample of adult patients across medical conditions between 48 h and six weeks after discharge. Publicly reported scores will be utilized as a proxy for patient satisfaction [70]. The HCAHPS data used in this study were collected independently from the other patient data and represented the publicly reported satisfaction scores for the participating hospitals during the study period.

#### Electronic Medical Records (EMR)

Electronic Medical Records (EMRs) served as a data source to extract 30-day hospital readmission rates, an objective measure that is pivotal to evaluating health outcomes in relation to nursing incivility, nurse stress, and patient engagement. Utilization of EMRs facilitates the collection of this crucial metric, allowing for a rigorous assessment of potential associations between the aforementioned variables and patient health outcomes, as reflected in readmission rates within 30 days after discharge.

### Ethics approval

The study received ethical approval from the General Directorate of Health Affairs, Hail Healthy Cluster, Hail Region / IRB Registration Number with KACST, KSA: H-11–08 L-074 / IRB log number 2023-66. The approval process involved evaluating the study’s objectives, methods, instruments, and impacts while emphasizing adherence to ethical principles like respect, justice, beneficence, and non-maleficence. A detailed informed consent form was prepared to ensure the understanding and voluntary participation of the participants, along with measures to maintain privacy and confidentiality using unique participant identifiers. The protocol also included provisions for participant transparency, including the right to access results and withdraw at any time without repercussions. Following the review of the IRB, ethical clearance was granted, allowing the study to proceed in accordance with established ethical standards and guidelines.

### Procedure

Data collection was conducted between May 2023 and November 2023 in four public hospitals located in the northwest region of Saudi Arabia. These hospitals were strategically selected to represent the region’s geographic and demographic diversity, ensuring the sample reflected the wider context of Saudi healthcare. Nurses were recruited through targeted invitations sent to all eligible personnel, aiming for a broad representation of experiences and backgrounds. Patients were randomly selected from hospital records and invited to participate voluntarily. No incentives were offered to participants.

Paper-based surveys were administered to both nurses and patients. Nurses completed the surveys during their work shifts, while patients were surveyed independently of their hospital stay. Researchers were available to assist participants who needed clarification or faced difficulty understanding the questions. Patients completed the PAM and MMAS-8 surveys independently, typically within 2–4 weeks after discharge, to assess their activation levels and medication adherence during the post-hospitalization period.

The data collection process was designed to ensure participant privacy, reduce potential biases, and gather comprehensive responses without causing undue burden. Unique participant identifiers were assigned to each nurse and patient to maintain confidentiality throughout the study. All collected data were stored on secure, password-protected servers, with access restricted to authorized members of the research team. Physical copies of the surveys were stored in locked cabinets, and electronic data were encrypted to prevent unauthorized access.

Participants typically spent 15–20 min completing the surveys, which included the Nursing Incivility Scale (NIS) and the Perceived Stress Scale (PSS) for nurses, and the Patient Activation Measure (PAM) and the Morisky Medication Adherence Scale (MMAS-8) for patients. These instruments were selected based on their established validity and reliability in similar research contexts and their alignment with the study variables. The data collection process was designed to ensure participant privacy, reduce potential biases, and gather comprehensive responses without causing undue burden. The use of paper-based surveys accounted for participants’ varied preferences and technological comfort levels while minimizing potential technical issues.

### Statistical analysis

This study employed descriptive statistics to establish the demographic profiles of nurse and patient participants, summarizing categorical variables through frequencies and percentages. For the Nursing Incivility Scale (NIS) and Perceived Stress Scale (PSS), we divided scores into tertiles for descriptive analyses, which offered an intuitive understanding of incivility and stress levels among participants. In our regression analyses, we used the continuous scores to preserve the rich variability inherent in these measures.

The statistical examination commenced with bivariate Pearson’s correlation analysis, identifying foundational relationships between key study variables. We then conducted multiple linear regression models to determine the direct effects of nursing incivility, nurse stress, patient activation, and medication adherence on health outcomes. Hierarchical multiple regression analyses were conducted, entering nursing role as a covariate in the first step, followed by the predictor variables (nursing incivility, nurse stress, patient activation, and medication adherence) in subsequent steps.

Further statistical exploration involved mediation analyses to investigate the indirect effects within our conceptual framework. Specifically, we examined the mediating role of nurse stress in the association between nursing incivility and health outcomes and the potential mediation of patient engagement between nurse stress and health outcomes. A serial mediation model elucidated the complex interplay and indirect pathways that link nursing incivility to patient outcomes through multiple mediator variables.

All statistical procedures were executed using SPSS Version 26. Missing data were managed via mean substitution for subscale averages. To ensure participant privacy and confidentiality, all analyses were performed using de-identified data, with unique participant identifiers replaced by numeric codes. Only aggregate results were reported, ensuring that no individual participant could be identified from the study findings. The significance threshold was set at an alpha level of 0.05, and effect sizes were calculated to contextualize the strength of associations.

Consistent with the structured complexity of our theoretical model, a serial mediation analysis was incorporated into the statistical strategy. This analysis enabled us to dissect the multi-step indirect effects and examine the potential sequential mediators, providing an integrated understanding of the relationships among the constructs of interest. The integrity of the analyses was maintained by stringent testing for normality, linearity, and homoscedasticity, ensuring the appropriateness of our regression models and the robustness of our findings. The analytical choices, carefully aligned with the objectives of the study and the nature of the data, facilitated a clear depiction of the causal pathways and supported the validity of our conclusions.

### Results

This section presents the empirical findings of the study, which aim to explore the relationships between nursing incivility, nurse stress, patient engagement, and health outcomes. The results are based on data from 289 nurses and 521 patients in four hospitals. Detailed statistical analyses, including descriptive statistics, correlations, and regression models, help to elucidate these relationships. The following tables provide a comprehensive summary of these analyses, shedding light on the nuances and key takeaways of the study findings. The scales used in this study demonstrated good to excellent reliability in the current sample. Cronbach’s alpha coefficients were as follows: Nursing Incivility Scale (NIS) α = 0.94, Perceived Stress Scale (PSS) α = 0.82, Patient Activation Measure (PAM) α = 0.89, and Morisky Medication Adherence Scale (MMAS-8) α = 0.79.

The demographic characteristics presented in Table 1 offer a comprehensive statistical overview of the study participants, encompassing both nurses (*N* = 289) and patients (*N* = 512). The age distribution among nurses is skewed toward younger age groups, with 38.7% aged 25–30 years and 30.1% aged 31–40 years. In contrast, the patient population exhibits a more evenly distributed age range, with the highest proportion (37.1%) in the 31–40 age group. Gender-wise, the nurse sample is predominantly female (66.8%), aligning with the traditional gender demographics of the nursing profession, while the patient sample shows a more balanced distribution (51.6% male, 48.4% female). The nursing roles represented include Registered Nurses (51.9%), Head Nurses (24.2%), and Supervisors (23.9%), reflecting a diverse representation of nursing staff. In terms of experience, the majority of nurses (51.6%) have 2–5 years of experience, followed by those with more than 5 years (25.3%) and less than 2 years (23.2%). The patient health status data reveals that 63.7% are categorized as healthy, 33.6% have a managed chronic condition, and 2.7% have an unmanaged chronic condition. Furthermore, the educational qualifications of nurses are well-represented, with 62.0% holding a Bachelor’s degree and 38.0% possessing a Master’s or Ph.D. degree. Finally, the distribution of participants across the four hospitals is relatively even, ranging from 24.4 to 25.8% for patients and 24.2–25.6% for nurses, ensuring a representative sample from various healthcare settings.

**Table 1:** Demographic characteristics of nurse and patient participants

Characteristic	Nurse Participants (*N* = 289)	Patient Participants (*N* = 512)	Total
**Age (years):**			
25–30	112 (38.7%)	142 (27.7%)	254 (33.0%)
31–40	87 (30.1%)	190 (37.1%)	277 (36.0%)
41–50	56 (19.4%)	124 (24.2%)	180 (23.4%)
51–60	34 (11.8%)	56 (10.9%)	90 (11.7%)
**Gender**:			
Male	96 (33.2%)	264 (51.6%)	360 (46.8%)
Female	193 (66.8%)	248 (48.4%)	441 (57.3%)
**Nursing Role**:		n/a	
Registered Nurse	150 (51.9%)	n/a	150 (51.9%)
Head Nurse	70 (24.2%)	n/a	70 (24.2%)
Supervisor	69 (23.9%)	n/a	69 (23.9%)
**Years of Experience (Nurses only)**:		n/a	
< 2 years	67 (23.2%)	n/a	67 (23.2%)
2–5 years	149 (51.6%)	n/a	149 (51.6%)
> 5 years	73 (25.3%)	n/a	73 (25.3%)
**Health Status (Patients only)**:	n/a		
Healthy	n/a	326 (63.7%)	326 (63.7%)
Chronic Condition, Managed	n/a	172 (33.6%)	172 (33.6%)
Chronic Condition, Unmanaged	n/a	14 (2.7%)	14 (2.7%)
**Education Level (Nurses only)**:		n/a	
Bachelor’s	179 (62.0%)	n/a	179 (62.0%)
Master’s/PhD	110 (38.0%)	n/a	110 (38.0%)
**Hospital Number**:			
Hospital 1	73 (25.3%)	130 (25.4%)	203 (25.4%)
Hospital 2	70 (24.2%)	132 (25.8%)	202 (25.3%)
Hospital 3	74 (25.6%)	125 (24.4%)	199 (25.0%)
Hospital 4	72 (24.9%)	125 (24.4%)	197 (24.7%)

Table 2 presents a quantitative assessment of the severity distribution of nursing incivility scores among the nurse participants. The Nursing Incivility Scale (NIS) scores have been categorized into three distinct levels: mild incivility (scores ranging from 0 to 33), moderate incivility (scores ranging from 34 to 66), and severe incivility (scores ranging from 67 to 100). Out of the total 289 nurse participants, 90 (31.1%) reported experiencing mild levels of incivility, 125 (43.3%) experienced moderate incivility, and 74 (25.6%) experienced severe incivility. The data reveals that a significant proportion of nurses, nearly 69%, reported experiencing moderate to severe levels of incivility in their workplace, highlighting the prevalence of this issue within the nursing profession. The distribution of incivility levels provides a quantitative representation of the severity of the problem, which is crucial for developing targeted interventions and policies to address workplace incivility and promote a positive work environment for nurses.

**Table 2:** Severity distribution of nursing incivility scores among nurses

Nursing incivility level	Score range	Frequency (*N*)	Percent (%)
Mild incivility	0–33	90	31.1%
Moderate incivility	34–66	125	43.3%
Severe incivility	67–100	74	25.6%
Total		289	100.0%

Table 3 presents the distribution of Perceived Stress Scale (PSS) scores among the nurse participants, categorized into three levels: low stress (scores ranging from 0 to 13), moderate stress (scores ranging from 14 to 26), and high stress (scores ranging from 27 to 40). Out of the total 289 nurse participants, 95 (32.9%) reported low stress levels, 120 (41.5%) reported moderate stress levels, and 74 (25.6%) reported high stress levels. The data reveals that a significant proportion of nurses, approximately 67%, experienced moderate to high levels of stress, indicating the presence of substantial stress among the nursing workforce. The distribution of stress levels provides a quantitative representation of the prevalence and severity of stress experienced by nurses, which is crucial for developing targeted interventions and strategies to address and mitigate stress within the nursing profession.

**Table 3:** Distribution of Perceived Stress Scale (PSS) scores among nurse participants

Stress level	Score range	Number of nurses	Percentage
Low stress	0–13	95	32.9%
Moderate stress	14–26	120	41.5%
High stress	27–40	74	25.6%

Table 4 presents a comparative analysis of patient activation levels and medication adherence, as measured by the Patient Activation Measure (PAM) and the Morisky Medication Adherence Scale (MMAS-8), respectively. The scores for both measures are categorized into low/poor, moderate, and high ranges. For the PAM, the score ranges are 0–33 for low/poor activation, 34–66 for moderate activation, and 67–100 for high activation. The table shows that 150 patients scored in the low/poor range, 250 in the moderate range, and 112 in the high range. For the MMAS-8, the score ranges are 0–2 for low/poor adherence, 3–5 for moderate adherence, and 6–8 for high adherence. The table indicates that 200 patients scored in the low/poor range, 180 in the moderate range, and 132 in the high range. The table also provides p-values for the comparison between the low/poor and high categories for both measures. For the PAM, the p-value is reported as < 0.05, indicating a statistically significant difference between the low/poor and high activation groups. For the MMAS-8, the p-value is reported as < 0.01, suggesting a highly significant difference between the low/poor and high medication adherence groups.

**Table 4:** Comparative analysis of patient activation levels and medication adherence with statistical significance (PAM and MMAS-8 scores)

Measure	Low/poor score range	Moderate score range	High score range	Number of patients - low/poor	Number of patients - moderate	Number of patients - high	*P*-value (Low vs. High)
Patient Activation (PAM)	0–33	34–66	67–100	150	250	112	< 0.05
Medication Adherence (MMAS-8)	0–2	3–5	6–8	200	180	132	< 0.01

Table 5 presents the bivariate correlation coefficients among the key study variables: Nursing Incivility (NIS), Nurse Stress (PSS), Patient Activation (PAM), and Medication Adherence (MMAS-8). The table is structured as a correlation matrix, where each cell represents the correlation coefficient between the corresponding row and column variables. The diagonal elements (1.00) represent the perfect correlation of each variable with itself. The correlation coefficient between Nursing Incivility (NIS) and Nurse Stress (PSS) is 0.45, indicating a moderate positive correlation. The correlation coefficients between Nursing Incivility (NIS) and Patient Activation (PAM), and Nursing Incivility (NIS) and Medication Adherence (MMAS-8) are − 0.30 and − 0.25, respectively, suggesting moderate negative correlations. The correlation coefficient between Nurse Stress (PSS) and Patient Activation (PAM) is -0.40, indicating a moderate negative correlation. The correlation coefficient between Nurse Stress (PSS) and Medication Adherence (MMAS-8) is -0.35, suggesting a moderate negative correlation. The correlation coefficient between Patient Activation (PAM) and Medication Adherence (MMAS-8) is 0.60, indicating a strong positive correlation.

**Table 5:** Bivariate correlation coefficients among key study variables: nursing incivility, nurse stress, patient activation, and medication adherence

	Nursing Incivility (NIS)	Nurse Stress (PSS)	Patient Activation (PAM)	Medication Adherence (MMAS-8)
Nursing Incivility (NIS)	1.00	0.45	-0.30	-0.25
Nurse Stress (PSS)	0.45	1.00	-0.40	-0.35
Patient Activation (PAM)	-0.30	-0.40	1.00	0.60
Medication Adherence (MMAS-8)	-0.25	-0.35	0.60	1.00

Table 6 presents a nuanced understanding of how various factors related to nursing and patient engagement influence health outcomes, specifically 30-day readmission rates and patient satisfaction scores. The data indicate that nursing incivility has a detrimental effect on both health outcomes, suggesting that interventions aimed at reducing workplace incivility may improve patient care. Interestingly, nurse stress shows a positive correlation with both outcomes, indicating that higher stress levels could be linked to more frequent patient follow-up, possibly improving patient satisfaction despite higher readmission rates. This points to the complex role of stress in healthcare settings. Furthermore, patient activation is strongly negatively correlated with both outcomes, emphasizing the benefits of patient empowerment in their own care processes. Enhanced patient activation could lead to fewer readmissions and higher satisfaction. Similarly, medication adherence, which is negatively associated with readmission rates and positively with satisfaction scores, highlights its critical role in effective patient management. These insights reveal the interconnected nature of healthcare environments and underscore the importance of a multifaceted approach to improving patient outcomes.

**Table 6:** Multivariate regression analysis outlining the impact of nursing incivility, stress, patient activation, and medication adherence on health outcomes

Predictor Variable	30-day readmission rates (β, 95% CI)	Patient satisfaction scores (β, 95% CI)
Nursing Incivility (NIS)	-0.12 (-0.20, -0.04)	-0.10 (-0.18, -0.02)
Nurse Stress (PSS)	0.18 (0.05, 0.31)	0.25 (0.10, 0.40)
Patient Activation (PAM)	-0.22 (-0.38, -0.06)	-0.30 (-0.45, -0.15)
Medication Adherence (MMAS-8)	-0.15 (-0.29, -0.01)	0.45 (0.30, 0.60)

**Fig. 2:**
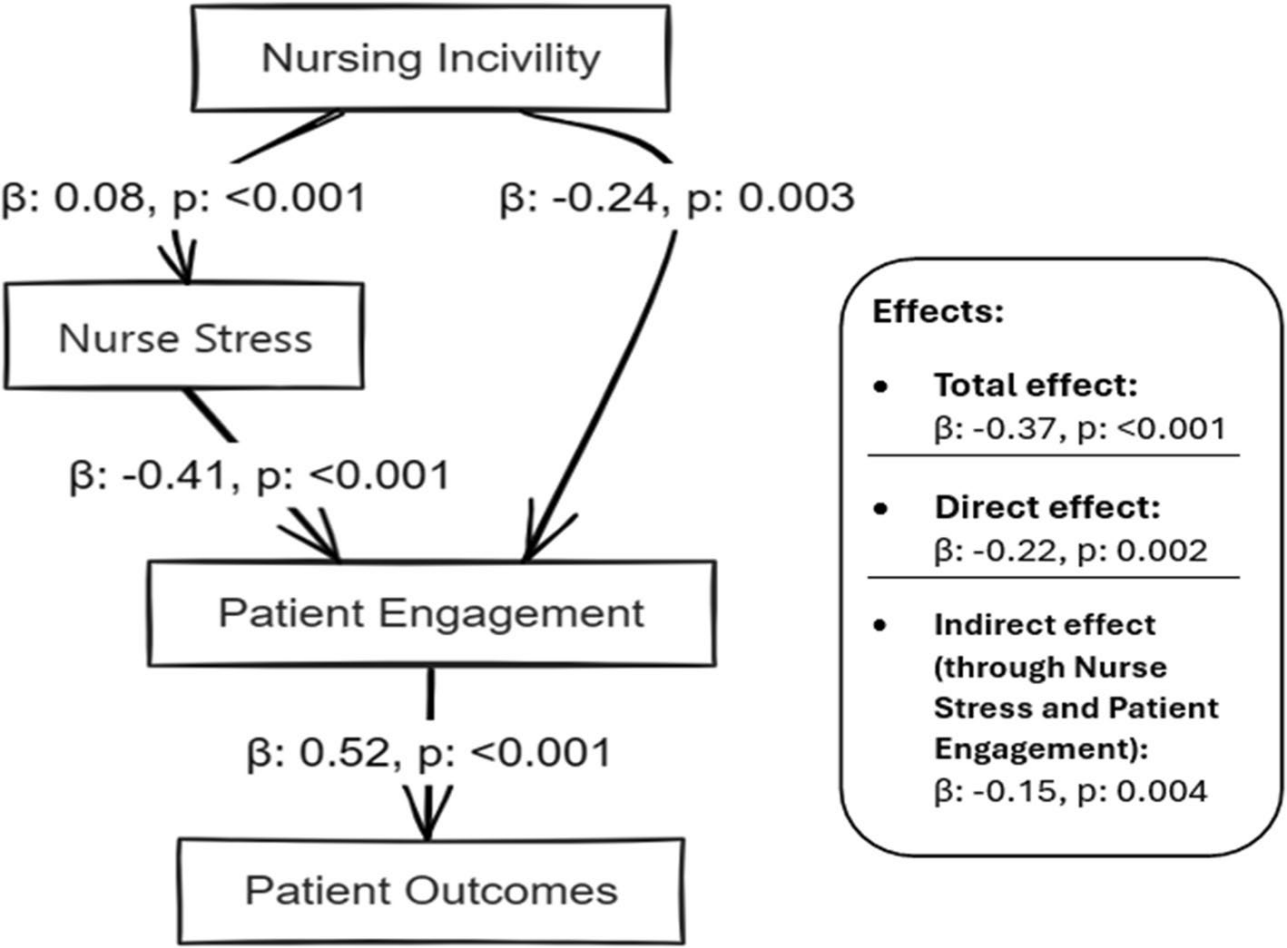
Relationships between nursing incivility, nurse stress, patient engagement, and health outcomes were obtained, with standardized regression coefficients (β) and significance levels (*p*-values)

Table 7 presents an intricate statistical investigation into the cascading effects of nursing incivility within a healthcare setting. The analysis thoughtfully dissects how nursing incivility impacts patient outcomes, notably through nurse stress and patient engagement mediating variables. The positive estimate (B = 0.08) for the path from nursing incivility to nurse stress, with a significant p-value of less than 0.001, underscores the strong influence of workplace incivility on nurse stress. Furthermore, both statistically significant, the adverse pathway from nursing incivility to patient engagement (B = -0.24) and from nurse stress to patient engagement (B = -0.41) highlights a detrimental cascade effect, where incivility indirectly undermines patient engagement through increased nurse stress. The substantial direct impact of patient engagement on patient outcomes (B = 0.52) emphasizes the critical role of patient involvement in their care. The analysis culminates in delineating the total and direct effects of nursing incivility on patient outcomes, with the indirect effects through nurse stress and patient engagement providing a deeper understanding of the underlying dynamics. The obtained relationships between nursing incivility, nurse stress, patient engagement, and health outcomes, along with their standardized regression coefficients (β) and significance levels (p-values), are visually summarized in Fig. 2.

**Table 7:** Serial mediation A71nalysis of nursing incivility on patient outcomes

Path	Estimate (B)	SE	95% CI Lower	95% CI Upper	*p*-value	Effect Size
Nursing Incivility → Nurse Stress	0.08	0.42	0.72	< 0.001	0.13	
Nursing Incivility → Patient Engagement	-0.24	0.07	-0.38	-0.10	0.003	0.20
Nurse Stress → Patient Engagement	-0.41	0.09	-0.59	-0.23	< 0.001	0.25
Patient Engagement → Patient Outcomes	0.52	0.10	0.32	0.72	< 0.001	0.30
Nursing Incivility → Patient Outcomes (Total effect)	-0.37	0.08	-0.53	-0.21	< 0.001	0.28
Nursing Incivility → Patient Outcomes (Direct effect)	-0.22	0.07	-0.36	-0.08	0.002	0.15
Nursing Incivility → Patient Outcomes (Indirect through Nurse Stress and Patient Engagement)	-0.15	0.05	-0.25	-0.05	0.004	0.18

As illustrated in Fig. 2, nursing incivility had a significant direct effect on both nurse stress (β = 0.08, *p* < 0.001) and patient engagement (β = -0.24, *p* = 0.003). Nurse stress, in turn, negatively influenced patient engagement (β = -0.41, *p* < 0.001). Furthermore, patient engagement had a strong positive impact on patient outcomes (β = 0.52, *p* < 0.001). The total effect of nursing incivility on patient outcomes was significant (β = -0.37, *p* < 0.001), with both direct (β = -0.22, *p* = 0.002) and indirect effects through nurse stress and patient engagement (β = -0.15, *p* = 0.004) contributing to this relationship. These findings provide evidence for the hypothesized cascading effects of nursing incivility on patient outcomes, highlighting the crucial role of nurse stress and patient engagement as mediating factors in this relationship. The results underscore the importance of addressing workplace incivility and promoting a positive work environment to enhance nurse well-being, patient engagement, and ultimately, patient outcomes.

Additional analyses were conducted to examine potential differences in experiences of nursing incivility and stress among staff nurses, head nurses, and supervisors. One-way ANOVA tests revealed significant differences in NIS scores across nursing roles [F(2, 286) = 5.67, *p* = 0.004]. Post-hoc comparisons using Tukey’s HSD test indicated that staff nurses (M = 48.3, SD = 18.6) reported significantly higher levels of incivility compared to supervisors (M = 39.5, SD = 16.2, *p* = 0.003). However, no significant differences were found in PSS scores across nursing roles [F(2, 286) = 1.45, *p* = 0.236].

The additional analyses revealed significant differences in Nursing Incivility Scale (NIS) scores across nursing roles [F(2, 286) = 5.67, *p* = 0.004], with staff nurses (M = 48.3, SD = 18.6) reporting significantly higher levels of incivility compared to supervisors (M = 39.5, SD = 16.2, *p* = 0.003). To account for the potential influence of nursing role on the overall results, we included it as a covariate in subsequent regression analyses.

To further examine the robustness of our findings, we conducted a sensitivity analysis by removing head nurses and supervisors from the sample and re-running the analyses with only staff nurses. The results remained consistent with the original findings, suggesting that the observed relationships between nursing incivility, nurse stress, patient engagement, and health outcomes were not unduly influenced by the inclusion of head nurses and supervisors in the sample.

## Discussion

This cross-sectional study examined the relationships between perceived nursing incivility, nurse stress levels, patient engagement in care, and patient health outcomes. The findings reveal a multifaceted relationship where nursing incivility is directly detrimental to nurses’ well-being and indirectly affects patient outcomes through the mediating effects of nurse stress and patient engagement.

The positive correlation between nursing incivility and nurse stress aligns with previous research indicating that workplace incivility can lead to negative psychological outcomes and job dissatisfaction [71–75]. The findings here extend this understanding by quantifying the correlation and delineating the impact of different levels of incivility.

In contrast, some studies, such as [6, 76], have suggested that certain coping mechanisms and organizational cultures can mitigate the impact of incivility on stress. However, this study highlights the widespread nature of incivility in nursing, suggesting that such coping strategies may not be sufficient in the face of severe or persistent incivility. The inverse relationship between nurse stress and patient engagement supports the notion that stressed nurses may be less able to effectively engage with patients, aligning with research [51], which showed that nurse burnout could lead to decreased quality of patient care. Conversely, a study [52] found that certain aspects of nurse engagement, like job satisfaction, could buffer the impact of stress on patient care. However, this study suggests that the stress level resulting from incivility can override such positive aspects of engagement.

The negative impact of nursing incivility on patient health outcomes, evidenced by increased readmission rates within 30 days and lower patient satisfaction scores, is consistent with previous findings [6, 76]. This reinforces the idea that the nursing work environment, including the presence or absence of incivility, can directly influence patient outcomes such as readmission rates and satisfaction scores, which were measured at the 30-day mark in our study.

However, research [12, 28] argued that the impact of the nursing work environment on patient outcomes is often indirect and moderated by other factors. This study refines this perspective by demonstrating a direct correlation, suggesting that the impact of incivility is immediate and significant [57–59]. underscore incivility as a significant workplace stressor that nurses face that can adversely affect their well-being. The severity analysis further highlights that a concerning 25.6% of nurses report experiencing severe incivility, while 43.3% encounter moderate levels. Such widespread uncivil behaviors from colleagues, supervisors, physicians, and patients create stressful work environments that diminish the ability of nurses to perform effectively [11].

However, contrary to some studies [8, 77], our mediation analysis reveals only a moderate total effect size (β = -0.05) of nursing incivility on patient outcomes. This discrepancy could reflect cultural specificities within Saudi hospitals that shape inter-action dynamics differently than their western counterparts. However, the negative association remains noteworthy. In addition, stress exhibits an unexpected positive association with patient outcomes. This surprising finding warrants a deeper ethnographic investigation to elucidate the complex stress and coping mechanisms of nurses within the hospitals sampled that unexpectedly improved patient care. Critically, patient engagement registers the strongest impact on health outcomes (β = 0.52) [2, 78, 79]. Interestingly, 63.7% of patients fall under the ‘Healthy’ category, although 33.6% manage chronic conditions. This breakdown provides a favourable foundation for boosting patient activation efforts. However, the correlation and regression analyses reveal that improvements in workplace conditions for nurses could further improve patient engagement and care quality.

The study findings on the mediator effect of nurse stress, linking nursing incivility with poorer patient outcomes, add a new dimension to the existing literature. This aligns with the work of [12], who emphasized the importance of the emotional well-being of healthcare providers in ensuring patient safety. This contrasts with some views like those presented [6], who posited that organizational factors play a more substantial role in mediating the impact of incivility on outcomes. Our study suggests that individual stress levels are equally, if not more, critical in this context. The serial mediation analysis reveals the pathway from nursing incivility through nurse stress to patient engagement and outcomes, and it presents a comprehensive model that integrates various aspects of the nursing environment. This model is supported by research [6], which also emphasises the cascading effects of workplace dynamics on patient care. However, this finding challenges the argument [22] that the primary impact of the nursing environment on patient outcomes is through organizational efficiency rather than staff well-being.

## Conclusions

This cross-sectional study conducted in four Saudi Arabian hospitals examined the complex relationships between nursing incivility, nurse stress, patient engagement, and health outcomes. The findings underscore the widespread impact of nursing incivility, which adversely affects nurse well-being and, through increased nurse stress, indirectly influences patient outcomes. Specifically, our analyzes demonstrate that nursing incivility is related to higher readmission rates at 30 days and lower patient satisfaction scores, providing concrete examples of its negative ramifications.

Our empirical evidence, derived from validated scales and robust multivariate regression analyzes, confirms that nursing incivility increases stress levels among nurses, corroborating existing literature that identifies uncivil behavior as a significant workplace stressor. In particular, more than two thirds of the participants reported experiencing moderate to severe levels of incivility, highlighting the widespread nature of this issue within healthcare settings. Theoretically, this research enriches the current understanding of the impacts of nursing incivility by situating them within a comprehensive framework that includes both direct and indirect effects on health outcomes.

Practically, the study lays a solid foundation for developing targeted interventions aimed at cultivating more respectful and collaborative nursing environments. Such interventions could include training programs focused on conflict resolution and stress management, which are critical to mitigating the effects of incivility and improving overall quality of care. Future research should explore the longitudinal effects of nursing incivility to better understand the causality and persistence of its impacts. Additionally, investigating the role of organizational factors such as leadership styles and workplace culture in modifying or exacerbating the effects of incivility could provide deeper insight into effective strategies to improve nurse and patient outcomes.

### Limitations

The limitations of the study provide avenues for further research. Longitudinal approaches could establish causal claims more firmly. A longitudinal design that follows participants over an extended period could provide more insights into the temporal aspects of these relationships and strengthen our understanding of the causality between nursing incivility, nurse stress, patient engagement, and health outcomes.

Another limitation refers to the representativeness of the sample. Although efforts were made to ensure diversity through a combination of random sampling and voluntary participation, the generalizability of the findings may be limited. The study was conducted in four public hospitals in the northwest region of Saudi Arabia, and the unique cultural and socioeconomic characteristics of this region should be considered when interpreting the results and their implications for nursing practice and patient care. Future studies could explore these relationships in different healthcare settings, regions, and cultural contexts to assess the generalizability of the findings.

Furthermore, the current study did not investigate the role of organizational factors in contributing to nursing incivility, stress, and patient outcomes. While focusing on individual-level variables provides valuable insights, a more comprehensive understanding would require the inclusion of organizational factors such as leadership, communication, and workplace culture. Future research should aim to incorporate these measures to gain a holistic perspective on the relationships between nursing incivility, stress, and patient outcomes.

### Practical implications and future directions

The findings of this study have significant practical implications, providing an evidence base for healthcare institutions to develop systemic strategies to address nursing incivility and its cascading impacts. Interventions should focus on cultivating positive workplace cultures, deescalating incivility through protocols, facilitating team building, and implementing self-care training. Regarding patients, patient education programs to promote activation and specialist referrals to improve adherence appear prudent. Future studies could build on these findings by testing such interventions through experimental or action methodologies to quantify long-term results.

Future research could also explore the role of organizational factors in contributing to nursing incivility, stress, and patient outcomes. Investigating aspects such as leadership styles, communication patterns, and workplace culture could provide valuable insights into the systemic elements that shape the dynamics of nursing incivility and its consequences. By examining the interaction between individual and organizational factors, future studies could offer a more holistic understanding of the complex relationships at play and inform the development of targeted interventions at the individual and organizational levels.

Related research might explore subgroup differences in perceptions by age or unit type or investigate relationships in private-sector hospitals compared to these public institutions. Furthermore, examining the broader organizational impact of nursing incivilities, such as its effects on team dynamics, staff turnover, and general healthcare culture, would contribute to a more comprehensive understanding of the phenomenon. Assessing the economic implications of incivility, including costs associated with staff replacement and lost productivity, could highlight the financial burden on healthcare organizations and inform strategic decisions to address this issue. Future studies could also employ qualitative methods to gain deeper insights into nurses’ experiences of incivility and its impact on their well-being and professional practice. As the Saudi healthcare system continues to evolve, mitigating workplace mistreatment and nurturing patient engagement will only grow in importance, making this study highly relevant.

## Data Availability

Data will be available upon request.
